# Evolving technology: the TRIFLO tri-leaflet mechanical valve without oral anticoagulation: a potential major innovation in valve surgery

**DOI:** 10.3389/fcvm.2023.1220633

**Published:** 2023-09-29

**Authors:** Thierry Carrel, Paul R. Vogt, Dominique Obrist, Hartzell Schaff

**Affiliations:** ^1^ Department of Cardiac Surgery, University Hospital Basel, Switzerland; ^2^ Hirslanden Clinic, Zürich, Switzerland; ^3^ ARTORG Center for Biomedical Research, University of Bern, Bern, Switzerland; ^4^ Mayo Clinic Rochester, Rochester, MN, United States

**Keywords:** aortic valve replacement, mechanical prosthesis, biological prosthesis, anticoagulation, particle velocytometry, tri-leaflet implant

## Abstract

The aortic valve is the most frequently diseased valve and aortic stenosis (AS) is the most prevalent valvular heart disease in developed countries. The diseased native aortic valve can be replaced by either a biological or mechanical valve prosthesis. The main concerns relate to durability, the need for oral anticoagulants and the incidence of complications related to this medication. Experimental, computational and biomolecular blood flow studies have demonstrated that the systolic forward flow but also the reverse flow phase at the end of the systole and leakage during the diastolic phase is mainly responsible for platelet activation and thrombosis. Better design of mechanical prosthetic heart valves must ensure smooth closing during flow deceleration and must eliminate high-shear hinge flow during diastole to prevent life-threatening thrombosis. A novel tri-leaflet valve should combine the favorable hemodynamics and the durability of existing mechanical heart valves and eliminate the less favorable characteristics, including the extremely rapid closing. In this paper, we discuss some issues of current mechanical heart valve prostheses and present a new valve design with the potential for significant innovation in the field. The TRIFLO Heart Valve, is a rigid, three-leaflet central flow heart valve prosthesis consisting of an alloyed titanium housing, and three rigid polymer (PEEK) cusps. This valve has a physiological operating mode. During the forward flow phase, the intraventricular pressure opens the leaflets so that blood can freely flow through with little obstruction, and with the deceleration of the blood flow, the leaflets close early and smoothly, minimizing blood flow regurgitation, blood cell damage, and activation of the coagulation cascade. Pre-clinical studies have shown pretty favorable results and a first-in-man study should start very soon.

## Introduction

Aortic valve stenosis is the most frequent heart valve disease. In the Cardiovascular Health Study that included 5,201 patients, a substantial increase in the prevalence of AS was observed with increasing age: 1.3% in patients aged between 65 and 75 years, 2.4% in those between 75 and 85 years, and 4% in patients older than 85 years (1). Untreated patients are exposed to significant risks and the overall prognosis of the disease is dismal with a mean survival estimated between 2 and 4 years after the diagnosis of a severe AS. The primary etiology is calcific degeneration of the valve cusps with narrowing of the valve's opening surface. Aortic regurgitation (AR) is less frequent and consists of diastolic reversal of blood flow from the aorta into the left ventricle, which may be due to aortic valve or aortic root disease. The prevalence of moderate and severe AR was estimated at 0.5% in the Framingham study (2). Treatment of AS and AR consists of aortic valve replacement (AVR), which reduces symptoms and improve long-term survival. For approximately 15 years, transcatheter aortic valve replacement (TAVR) has been used in older patients (>70–75 years) and those with considerable perioperative risk, but a general extension of the indications for TAVR is observed in intermediate and low-risk patients too (3–8). Primarily due to the assumption that patients receiving a tissue valve will be free of anticoagulant therapy, tissue valves have been extensively used in the last few years (an increase of 43,6% in the US over ten years and of 12.4% over two years in the EU) (9–12). In addition, adoption of TAVR has accelerated this trend.

## Historical background of mechanical valves

The caged-ball valve was developed and implanted in the 1960s and obstructed central flow. It was replaced by tilting-disc valves in 1970 and later Kalke and Lillehei introduced the concept of bileaflet valve at the University of Minneapolis (13). The low valve profile and the improved central flow had obvious hemodynamic advantages compared to the bulky cage-ball design, particularly in the mitral position; the prosthesis was then manufactured by St-Jude Medical and implanted for the first time in 1977. This valve made of pyrolytic carbon became the gold standard and more than two million valves have been implanted worldwide. Later, the ATS AP 360 prosthesis (Medtronic, Minneapolis, USA) and the On-X valve have been the only modest innovations regarding bi-leaflet valve design.

However, reducing the energy required to eject the blood through the valve did not eliminate the flow-induced activation of the coagulation cascade. This is the reason why current bi-leaflet mechanical valves still require life-long warfarin.

Fortunately, progress has been made to analyze better the relationship between flow characteristics and thromboembolic complications of mechanical heart valves. Experimental, computational and biomolecular blood flow studies have demonstrated that the reverse flow phase at the end of the systole and leakage flow during the diastolic phase is mainly responsible for platelet activation and thrombosis (14–18). Recent computational studies also highlighted the relevance of systolic forward flow on platelet activation (16, 18). Nevertheless, most of the medical community still believes that oral anticoagulation following implantation of a mechanical heart valve is required because foreign material is exposed to blood flow.

## Anticoagulant treatment following mechanical valve implantation: still a critical issue

According to the current guidelines, patients who receive a mechanical heart valve must undergo life-long oral anticoagulation using vitamin K antagonists (VKA), with warfarin being the most frequent treatment (9, 10). Indeed, VKAs are the only oral anticoagulants approved for this indication. Besides the narrow therapeutic window, major drawbacks are variable dose response in individuals, interaction with some foods and drugs, and variable laboratory monitoring. Patients who do not take anticoagulation undergo a high risk of valve thrombosis. This risk also exists when the international normalized ratio (INR) remains below the targeted 2.0–3.0 range for a prolonged period. On the other hand, major fluctuations may still occur despite strict adherence to higher INR values and lead to bleeding and thromboembolic complications that may cause considerable morbidity and mortality. This is one of the reasons why even younger patients may avoid VKAs and choose tissue valves despite their limited durability (11, 12).

In randomized studies and registries, new oral anticoagulants (NOACs) have demonstrated their superiority regarding reduced bleeding complications in patients with non-valvular atrial fibrillation compared to VKAs with similar efficacy and are recommended as first choice in the guidelines (19). Unfortunately, a simple translation of these results to patients with a mechanical valve is inaccurate, and NOACs are still not indicated for this special condition.

In the RE-ALIGN trial (clinicaltrials.gov number NTC 01505881), the use of dabigatran as compared with warfarin was associated with an increase of the composite of death, stroke, systemic embolism, and myocardial infarction (8% vs. 2%, respectively; *P* = 0.11), as well as bleeding complications (27% vs. 12%, respectively; *P* = 0.01). The trial was stopped prematurely (20). The small, pilot, phase 2 CATHAR trial investigated the safety and efficacy of rivaroxaban use in mechanical prostheses but was stopped due to low enrolment of patients (clinicaltrials.gov number: NCT02128841) (21).

The PROACT Xa trial (clinicaltrials.gov number: NCT04142658) investigated the On-X mechanical valve (On-X Life Technologies, Austin, Texas, USA) in 375 high-risk patients randomized at three months post-AVR to receive aspirin with oral anticoagulation for a targeted INR of 1.5–2.0 or 2.0–3.0 (22). Through 5-year follow-up, bleeding rates were significantly lower for patients in the low INR group (*P* = 0.002), without significant increases in thromboembolic events (*P* = 0.13).

Based on this study, the FDA approved warfarin management of selected aortic valve patients at INR levels between 1.5 and 2.0. The low-risk arm in which 201 patients ≥18 years of age without thromboembolic risk factors were randomized to receive a double antiplatelet aggregation treatment or standard warfarin plus aspirin was terminated for excess cerebral thrombo-embolic events (3.12% per patient-year vs. 0.29% per patient-year, *p* = 0.02) in the DAPT group with no differences in bleeding or all-cause mortality (23).

A study investigating 863 patients with an On-X valve who received either apixaban 2 × 5 mg daily or warfarin with a target INR between 2 and 3 (ClinicalTrials.gov number, NCT04142658) was stopped because of an excess of thromboembolic events in the apixaban group while bleeding events were similar in both groups (24).

Other studies have examined the value of self-monitoring to improve the quality of long-term anticoagulation with VKAs. Koertke and co-authors performed a randomized trial in which 2,673 patients undergoing SAVR were randomized to anticoagulation with a target INR of 1.8–2.8 vs. 2.5–4.5. After 2-year follow-up, neither the rate of thromboembolic events nor the rate of bleeding was significantly different in the low-dose vs. conventional dose groups (25).

In a meta-analysis of 11 randomized trials comparing self-monitoring and management of anticoagulation found a significant decrease of thromboembolic events in the self-monitoring group with similar rates of bleeding and death among 6,417 participants (26).

Another potentially important investigation, the DIAMOND trial (ClinicalTrials.gov number NCT05687448) is designed to determine if patients with mechanical AVR can be maintained effectively with a better safety (net clinical benefit) for apixaban compared to warfarin. In the study group, patients are treated with apixaban 5 mg twice daily while in the comparison group, patients receive warfarin with an INR target of 2.5 (range: 2.0–3.0). The primary objective is to demonstrate that antithrombotic treatment with apixaban is non-inferior to warfarin for the primary net clinical benefit endpoint of ischemic outcomes (death, myocardial infarction, stroke, systemic embolism and valve thrombosis) and bleeding. The study is about to receive public funding to start inclusion.

Finally, another aspect that may gain importance soon is the possibility of telemedicine-guided INR management to increase patient compliance, and improve oral anticoagulation quality. Some interesting trends have been observed during the pandemic while telemedicine and artificial intelligence may further boost such approaches (27–29).

## The main problems of traditional mechanical valves

**Figure 1:**
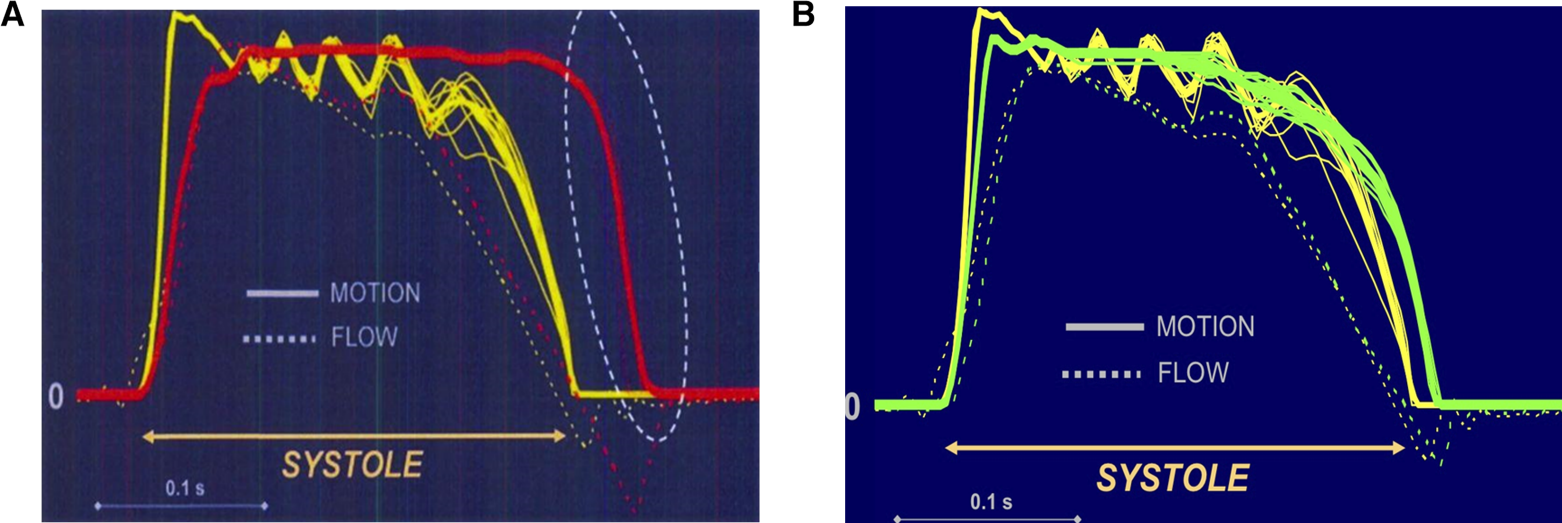
(**A**) Closing mode comparing a tissue valve (yellow) with a mechanical bi-leaflet valve (red). The St Jude valve is wide open at zero flow while the tissue valve (CE) is fully closed. Left is valve opening and right valve closure. The bi-leaflet valve cannot close without reverse flow because flow deceleration in late systole does not generate sufficient closing pressure. Unlike the leaflets of a native valve (or of a tissue valve), the carbon leaflets start closing only with the onset of reverse flow which accelerates rapidly due to the low pressure in the relaxing ventricle. Moreover, the valve closes extremely fast and exhibits leaflet rebound while reverse flow velocities as high as 200 m/sec (approximately 700 km/h) occur. (**B**) Closing mode comparing a tissue valve (yellow) with a mechanical tri-leaflet valve (green). The tri-leaflet valve closing mechanism is rather similar to that of a tissue valve (CE). Left is valve opening and right valve closure.

Interestingly, failures of mechanical valves in the past have always been related to high forces generated by the non-physiologic closing mode. These include fracture of the outlet strut (Bjork-Shiley) by closing rebounds (30, 31), leaflet erosion (Duromedic) by cavitation (32), thrombosis (Medtronic Parallel) (33, 34) and closing leaflet dysfunction (Medtronic Advantage) as well as by design flaws (35). The forces observed during a mechanical bi-leaflet valve's closure are by far higher than those needed to close a tissue valve. This type of valve cannot close without reverse flow because flow deceleration in late systole does not generate sufficient closing pressure (Figure 1); thus, the valve closes late extremely rapidly (36–38).

Excessive reverse flow velocities during this fast closing phase leads to extremely low pressure causing cavitation, which describes a phenomenon of “blood boiling”. Nitrogen and carbon dioxide are extracted from the blood and bubbles with a longer lifespan can be detected in the systemic circulation as “high-intensity transient signals” (39–41). Moreover, platelets are extremely sensitive to shear stress and instantaneously develop membrane tethers, leading to platelet aggregates (42). Exposure to unfavorable flow conditions or even stagnation promotes further thrombus growth. It has been found that shear stresses exceeding the threshold for platelet activation are present during the diastolic phase of the cardiac cycle.

After prosthetic valve closing, aortic blood pressure leads to a leakage flow through the narrow gaps in the hinge recesses of bi-leaflet valves. Like the non-physiologic closing mode, this leakage flow is suspected to lead to shear-induced thrombosis in the hinge region, which carries the risk of thromboembolism and leaflet immobilization (15, 17).

## Optimizing the design: potential advantages of a tri-leaflet mechanical valve

Better design of mechanical prosthetic heart valves must ensure smooth closing during flow deceleration and eliminate high-shear hinge flow during diastole to prevent life-threatening thrombosis.

A novel tri-leaflet valve should combine the favorable hemodynamics and the durability of existing mechanical heart valves and eliminate the less favorable characteristics, including the extremely rapid closing (37, 38, 42).

In vitro studies suggest the three valve cusps close at the onset of diastole within an average closing time of about 60 milliseconds, much slower than bi-leaflet valves and similar to the softer closing mode of a tissue valve (36, 38). Further studies of this tri-leaflet mechanical valve using micro-particle image velocimetry did not show critical regions of flow stagnation and zones of excessive shear in the pivoting region suggesting low potential for thrombogenic events that may allow for avoidance of long-term anticoagulation.

**Figure 2:**
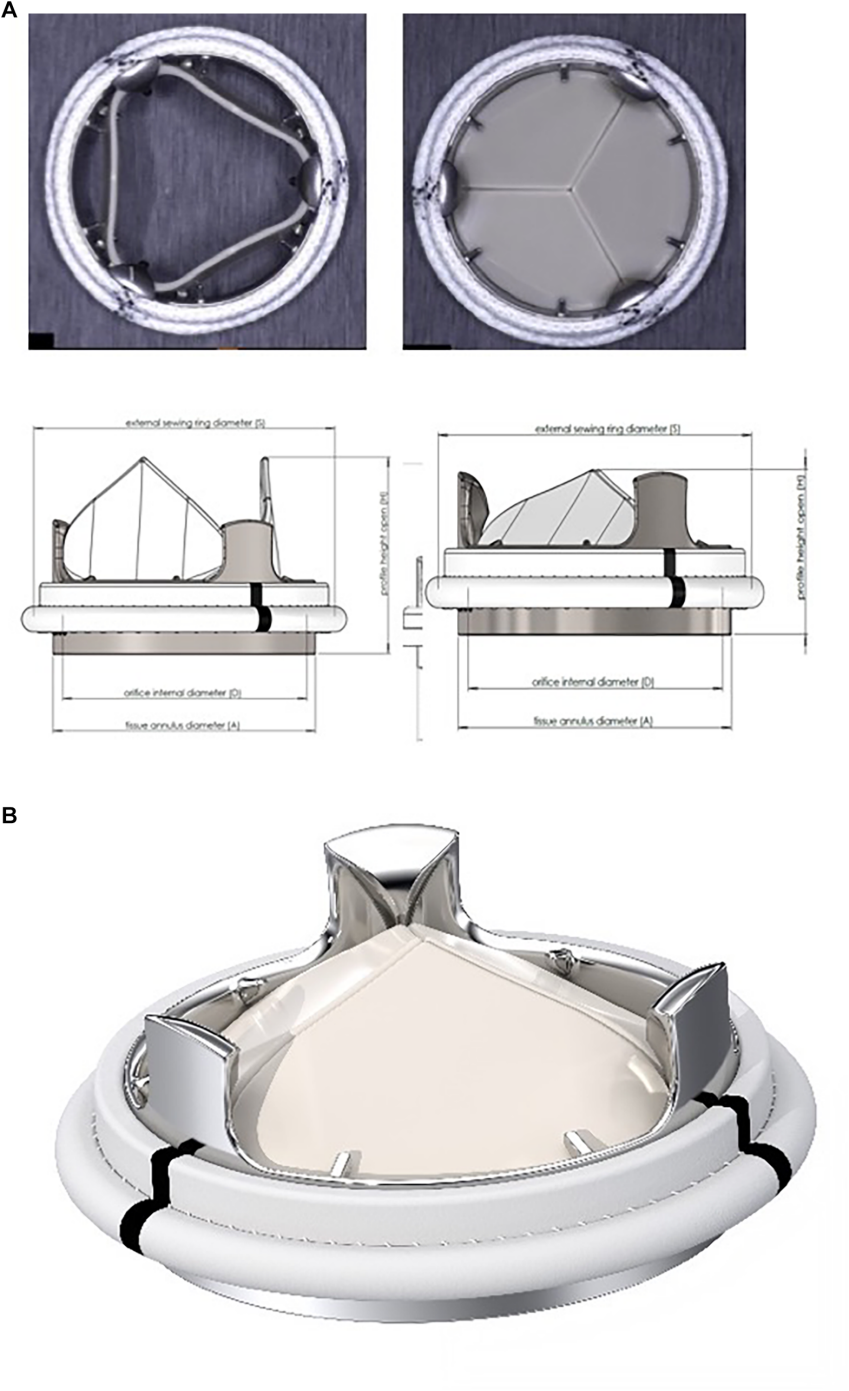
(**A**) The TRIFLO valve in the overview. (**B**) The TRIFLO valve with opened and closed leaflets.

The new tri-leaflet valve (designed originally by Didier Lapeyre, MD and called later the TRIFLO Valve, Novostia, Switzerland) is much more similar to a biological valve than to a bi- leaflet valve (Figure 2). The TRIFLO Heart Valve, is a rigid, three-leaflet central flow heart valve prosthesis consisting of an alloyed titanium housing, three rigid polymer (PEEK) cusps and a polyester sewing ring. Figure 2 shows the valve in the open and closed positions. Due to its configuration, the TRIFLO heart valve has a physiological operating mode. During the forward flow phase, the intraventricular pressure opens the leaflets so that blood can freely flow through with little obstruction, and with the deceleration of the blood flow, the leaflets close early and smoothly, minimizing blood flow regurgitation, blood cell damage, and activation of the coagulation cascade.

The housing and the leaflets are designed to guide and control leaflet motion, ranging from the open to fully closed position with a virtual pivot mechanism (no cavities or fixed points of rotation). Flow separation, blood stagnation areas and turbulent shear stresses, are minimized. The housing incorporates an orifice with flared inlet extending through the annulus, aimed at preventing pannus overgrowth on the inflow side and the flow turbulences, as well as a flared outlet to maintain optimal flow. The three leaflets are equally sized and shaped and are protected against impingement with one another by the three pivot guards on the outflow side of the housing.

Similarily to other valves, the sewing ring is knitted from velour polyester fabric. Three black reference markers facilitate orientation, the sewing ring is designed to be positioned supra-annularly (Figure 2).

## Preclinical data

Pre-clinical data on the TRIFLO Heart valve was obtained using animal implantation to demonstrate the safety and performance of the device. A regulatory animal study was conducted between January and July 2022 using the standard adult sheep model at the University of Minnesota. Six sheep were implanted with 21 mm TRIFLO Heart Valves, and two sheep were implanted with the control valves (On-X). All animals survived the 140-day minimum implant duration. Anticoagulation treatment consisted of heparin 2,000 IU twice daily for 2 days only.

**Figure 3:**
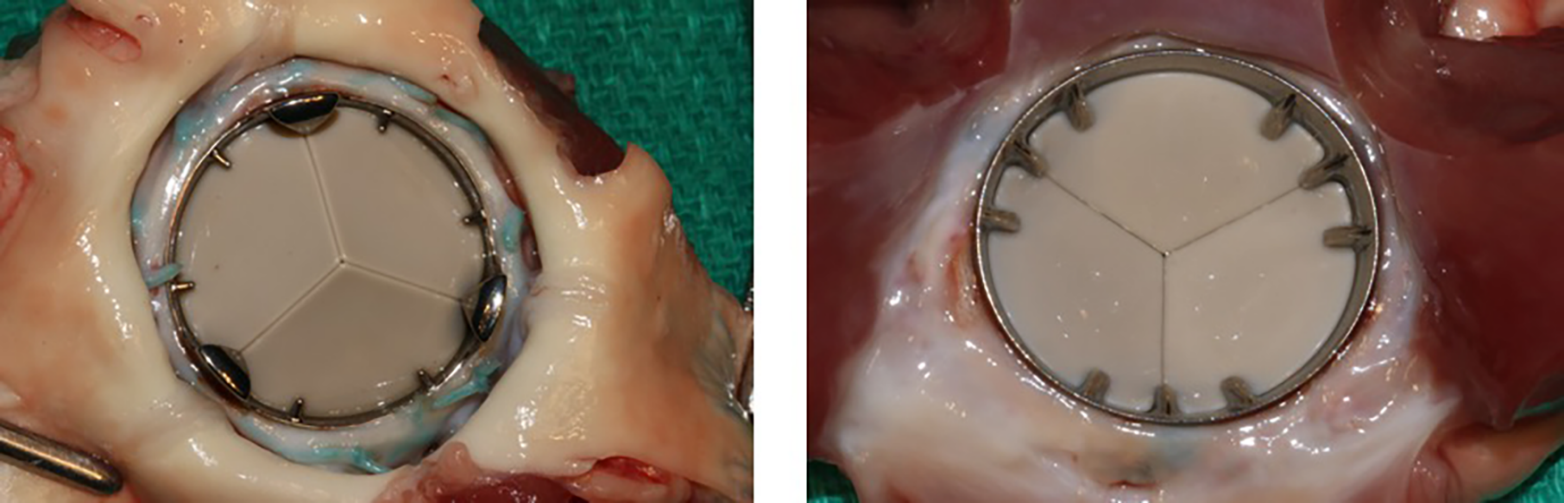
Specimen of TRIFLO valve after animal explantation show no thrombotic deposits on the leaflets from the aortic view (left) nor the internal ring nor excessive fibrous tissue formation (pannus) at the level of the sewing ring and/or below the valve on the ventricular side (right).

Serial post-operative blood samples showed no significant difference between the tested valves and no negative experimental device effect. The device hemodynamic parameters measured were normal and no surgical handling difficulties for the test valve were reported. Pathology studies revealed that the interior surfaces of the housing, the hinge region, and the leaflets were free of thrombus and pannus (Figure 3). The tissue surrounding the sewing ring demonstrated normal host reaction response, chronic inflammation and a good endothelialization. Post-mortem examination of the major organs was normal in all animals. All valves had normal surfaces after implant as observed with microscopic inspection and leaflets were clean at scanning electron microscopy inspection. Structural integrity evaluation revealed normal wear after five months of implantation and no sign of cavitation, corrosion or structural failure. Only implantation and long-term observation in humans will allow to confirm the favorable results observed in the animal studies, especially regarding the following expected clinical benefits:

• Avoidance of long-term anticoagulation and related bleeding complications and requirements for monitoring. We expect the percentage of major bleeding complications at 12 months with TRIFLO Heart Valve to be non-inferior to the percentage of major bleeding according to published data of the biological valve and lower than the values obtained with other similar mechanical valves requiring anti-coagulant treatment (43, 44).
• Elimination of iterative valve replacements due to the durability of the mechanical characteristics of the valve.
• Improvement in the overall quality of life measured by the KCCQ (Kansas City Cardiomyopathy questionnaire) (45) compared to current mechanical valves which require chronic anticoagulation

Although the TRIFLO Heart Valve has been extensively tested *in vitro*, by computer simulation and in animals studies, human studies are essential to validate device safety and performance. A lifelong durable valve without the need for anticoagulation has been the police of prosthetic heart valve development. The introduction of the TRIFLO valve in the clinical practice promises to be an important development in valve surgery. The first-in-man implantation that is planned for Q4 2023 has the potential to revolutionize valvular surgery, in a similar way that TAVR did more than 15 years ago.

Nevertheless, the residual risks following the implantation of the TRIFLO Heart Valve are not different from those associated with any surgical aortic valve implant. These risks include: endocarditis, major bleeding, myocardial infarction, new onset of atrial fibrillation, need for pacemaker implantation, paravalvular leak, stroke, thromboembolism, transient ischemic attack, and valve thrombosis.

The clinical investigational plan mandates careful patient selection by the investigator and the onsite Heart Team to minimize the risks for the subjects enrolled in this clinical trial. In addition, a centralized assessment of the subject by a Clinical Review Committee (CRC) will confirm subject eligibility through careful preoperative evaluation. Close postoperative monitoring will also minimize foreseeable risk and discomfort.

In addition, patients will be submitted to carotid Doppler examination to allow early detection of any microemboli (46). The patients will be examined under standard conditions: quiet room, supine position, eyes closed, and the start of the recording after resting for at least 10 min.

The heart rate is monitored during each examination. The Doppler frequency spectra of both carotid arteries (CAs) are recorded simultaneously and continuously for 30 min. The emitting power and the gain of the channels are set at the lowest intensity required to demonstrate a weak background flow velocity spectrum. This allows easy recognition of embolic signals, which appear as bright spots within the background spectrum. Emboli are identified by three different methods:

(1) Visually, on the monitor displaying the fast Fourier transform Doppler color-coded spectra of both CAs;
(2) Acoustically, by continuous on-line observation by the examiner using headphones; and
(3) Computer-assisted, using the system software (Emboli Detection Program). Embolic signals are identified by an experienced sonographer according to the following criteria:
  • They are short (<0.1 s), transient, unidirectional, high-amplitude signals, with a narrow spectrum;
  • They occur at random in the cardiac cycle and change their frequency/velocity depending on their location in the cardiac cycle and as they pass through the sample volume;
  • And they generate a chirping audio quality, with a harmonic tone.

Only signals detected acoustically and visually will be considered. For correlations, the emboli count in the left and right CAs are added to a sum score (FES during 30 min). For various reasons, the number of embolic signals per time unit is the most reliable parameter of the embolic signals recorded by TCD monitoring.

## Discussion

There has been little true innovation in the development of surgically implanted heart valve prostheses over the last four decades. Current generation mechanical valves have the disadvantages of long-term anticoagulation with warfarin, and stented xenograft bioprostheses, both porcine and pericardial, have limited durability. Indeed, much of the lack of development in mechanical valve substitutes relates to industry focus on transcatheter devices and the huge penetration of TAVR in clinical practice. Indeed, there is a major problem of under- or misinformation by clinicians and patients regarding the long-term consequences of TAVR implantation in patients with long life expectancy. Although transcatheter valves have dramatically improved the outlook of elderly patients with AS, there is little appreciation of actual long-term data and high TAVR explantation mortality rates. Clearly, there continues to be a huge unmet need for more durable and less thrombogenic heart valves worldwide, particularly for younger patients, including children and women of childbearing age suffering from congenital or rheumatic valve disease.

Patients living with current mechanical valves are estimated to have only a 50% chance of being alive without experiencing serious thromboembolic or bleeding complications (47). Tissue valves fail in the mid-term according to the age of the patients at the time of implantation. In developing countries, there are two main challenges: the long-term oral anticoagulation in patients with a mechanical prosthesis due to a lack of monitoring INR and the limited durability of tissue valves, particularly when implanted in younger patients (<40 years of age).

Almost six decades after the pioneering work of Albert Starr, Alain Carpentier, and Walton Lillehei, prosthetic heart valves are still based mainly on concepts of the 1970s. Today but also in the near future, there is and will be a global need for affordable and safe valve substitutes for younger patients, especially in developing countries (47–52). Repetitive interventions using only tissue valves are not optimal for these patients.

Older mechanical valve designs are still competing with the less durable technology of tissue valves due to a lack of interest in new valve designs and materials. Surgeons should express the need and willingness to use such new valves. Based on best-practice industrial design and test principles, the long-term performance of a valve prosthesis is *a priori* predictable, which is in contrast to any treatment to avoid xeno-tissue degeneration that requires years of follow-up before disadvantages can be ruled out.
